# The Effect of Salt-Processed* Psoralea corylifolia* on Generative Organ Targeting

**DOI:** 10.1155/2016/7484202

**Published:** 2016-10-20

**Authors:** Gen-hua Zhao, Cui-ping Yan, Zi-sheng Xu, Qian-qian Gao, Zhi-peng Chen, Wei-dong Li

**Affiliations:** ^1^College of Pharmacy, Nanjing University of Chinese Medicine, 138 Xianlin Avenue, Nanjing 210023, China; ^2^Jiangsu Jumpcan Pharmaceutical Group Co., Ltd., Taizhou 225441, China; ^3^Department of Chemistry, Hong Kong Baptist University, Kowloon Tong, Hong Kong; ^4^Engineering Center of State Ministry of Education for Standardization of Chinese Medicine Processing, Nanjing University of Chinese Medicine, Nanjing 210023, China

## Abstract

Psoralen and isopsoralen are two isomers and main effective components within* Psoralea corylifolia*. In order to investigate the salt-processing effect on tissue distribution characters of psoralen and isopsoralen, a sensitive and accurate ultrahigh pressure liquid chromatographic tandem mass spectrometric (UHPLC-MS/MS) method has been developed and validated for simultaneous determination of the 2 components in rats' tissues after administration of the extracts that came from either crude or salt-processed* Psoralea corylifolia* L. Data displayed that both areas under the curve (AUC) of psoralen and isopsoralen from salt-processed scurfpea fruit group were significantly increased compared with that of the crude herb group, especially in heart (*p* < 0.05), ovary, and testes (*p* < 0.001). Though the RE and RC_max_ of psoralen and isopsoralen in all of the investigated organs were over 1.0, generative organs kept the maximum value. The experiment manifested that salt-processing of scurfpea fruit can increase the distribution of psoralen and isopsoralen to generative organs, heart and spleen, and the distribution of psoralen and isopsoralen to generative organs is significantly higher compared to heart and spleen (*p* < 0.01). Results indicate that salt-processing of scurfpea fruit can significantly increase the distribution of psoralen and isopsoralen to generative organs.

## 1. Introduction

Meridian tropism theory, one of the basic theories in TCM, plays an important role to select herbs which are based on clinical syndromes [1]. According to this theory, herbs have special affinities to certain organs and channel systems of the body and exhibit special effects on diseases of these systems or organs [2, 3]. Herb processing technology, which is based on herb characteristics and clinical purpose, makes herbs more effective in clinical usage. There are various traditional methods to process herbs, such as salt-processing, frying with sand or oil, stewing by water or rice wine, and braising with rice wine [4]. According to TCM theory, these processing methods can increase the efficacy, reduce toxicity, alter the channel tropism of the original herbs, or improve herbs' smell and taste [5].

*Psoralea corylifolia* is the fruit of* Psoralea corylifolia* L., which belongs to Leguminosae. It was frequently been used in TCM prescription to prevent or treat various body disorders and diseases, such as osteoporosis and bone fracture [6], arthralgia, and asthma [7, 8]. More than 10 compounds were isolated from* Psoralea corylifolia*, such as psoralen, isopsoralen, psoralidin, bavachin, corylin, bavachalcone, and bakuchiol [9, 10].

Psoralen and isopsoralen are 2 furocoumarin compounds and the main effective components in scurfpea fruit. Recent studies demonstrated that psoralen and isopsoralen specifically showed estrogen-like activity [11, 12], osteoblastic proliferation accelerating activity [13, 14], anticancer activity [15], antibacterial activity [16], and so forth. However, to the best of authors' knowledge, no information was available about the tissue distribution character of the 2 compounds, and no investigation was reported comparing the fore-and-aft salt-process effect on scurfpea fruit.

In this study, a sensitive, accurate ultrahigh performance liquid chromatography tandem mass spectrometry (UHPLC-MS/MS) method was developed to determine psoralen and isopsoralen in rats' tissues. It was the first time to use targeting parameters to explain that salt-processing would change the special affinity of psoralen and isopsoralen to generative organs.

## 2. Materials and Methods

### 2.1. Materials and Reagents

**Figure 1:**
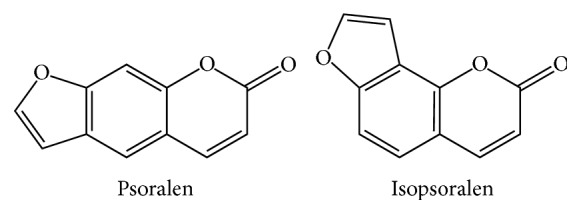
Chemical structures of psoralen and isopsoralen.

The herb scurfpea fruit was purchased from Nanjing Haichang Chinese Medicinal Decoction Pieces Factory (Nanjing, China). The authentication of the herb samples was conducted by Professor Jianwei Chen of Nanjing University of Chinese Medicine. Voucher specimens (number NJUTCM-20120327) were deposited at the Chinese medicinal herbarium of Nanjing University of Chinese Medicine (Nanjing, China). Psoralen (P), isopsoralen (IP), and scoparone (internal standard, IS) were obtained from Shanghai Usea Biotech (Shanghai, China). The purities of all the standards were over 99%. The chemical structures of psoralen and isopsoralen were presented in Figure 1.

Methanol (HPLC grade) was a product of Calepure Company Ltd. (Canada). Formic acid (HPLC grade) was purchased from Tianjin Damao Chemical Reagent Company. Milli-Q ultrapure water (Millipore, Bedford, MA, USA) was used in all relative analyses. All of the other relative reagents were of analytical grade.

### 2.2. UHPLC–MS/MS Instrument and Analytical Conditions

All separations were performed on a DGU-20A 5R series UHPLC system equipped with an LC-30AD binary pump (Shimadzu Corporation UFLC XR, Kyoto, Japan). Mass spectrometry was conducted by using a 5500 triple quadrupole tandem mass spectrometer equipped with an electrospray ionization (ESI) source (AB SCIEX, Foster City, CA, USA).

The chromatographic separation was achieved on Acquity UHPLC BEH C_18_ (100 mm × 2.1 mm, 1.7 *μ*m) column by employing Waters Acquity UHPLC system consisting of a binary solvent manager, a sample manager, and a column temperature controller (Waters Corp., Milford, MA, USA). The chromatographic conditions were recorded as follows: injection volume was 2 *μ*L; column temperature was maintained at 40°C; the mobile phase was composed of 1% formic acid (A) and acetonitrile (B) with gradient elution (0 → 1 min, 5%B → 50%B; 1 → 3 min, 50%B → 90%B; 3 → 6 min, 90%B → 90%B; 6 → 6.5 min, 90%B → 5%B) at a flow rate of 0.3 mL/min.

In order to achieve maximum sensitivity, a Waters Quattro Premier XE™ triple quadrupole mass spectrometry (Waters Corp., Milford, MA, USA) equipped with an ESI source was employed for detection. The mass spectrometry was operated in positive ionization mode and the ion spray voltage was set at 5.5 kV. The optimized parameters were recorded as follows: ion source temperature, 600°C; curtain gas (CUR), 35 PSI; ion source gas 1 (GAS1), 50 PSI; ion source gas 2 (GAS2), 60 PSI; ion spray voltage (IS), 5500 V; declustering potential (DP), 86.13 V; collision energy (CE), 30.22 V; entrance potential (EP), 6.53 V, and collision exit potential (CXP), 9.69 V. Multiple reaction monitoring (MRM) mode was applied for the quantitation at [M+H]^+^ *m/z* 187.2 →* m/z* 143.0 for psoralen and isopsoralen and at* m/z* 207.2 →* m/z* 151.1 for scoparone.

### 2.3. Preparation of Crude and Salt-Processed Scurfpea Fruit Extracts

Crude and salt-processed scurfpea fruits, 50.0 g for each, were soaked in 500 mL distilled water and decocted twice for 20 min per time. The decoctions were filtered and concentrated separately to get crude and salt-processed scurfpea fruit aqueous extracts, respectively, each with a concentration of 1 mL of concentrated decoction equivalent to 0.2 g of herbs and adding carboxymethylcellulose sodium to each decoction at a concentration of 0.2%. The final solutions were stored at 4°C refrigerator before use.

For analysis, 1 mL of each of the above final solutions was 10-fold diluted and filtrated before injection. The contents of psoralen and isopsoralen were quantified by using external standard method. Chromatographic condition was described above.

### 2.4. Preparation of Stock Solutions and Quality Control Samples

Stock solution was prepared by dissolving psoralen (4.63 mg) and isopsoralen (5.08 mg) in methanol (10 mL) at concentrations of 463 *μ*g/mL for psoralen and 508 *μ*g/mL for isopsoralen. Working solutions of psoralen and isopsoralen were prepared by serial dilution of the stock solutions with methanol. The stock solution of IS was prepared in methanol at 1000 *μ*g/mL and diluted in acetonitrile to obtain working solution at 300 ng/mL.

Calibration samples were prepared by spiking 300 *μ*L blank plasma with 9700 *μ*L working solutions to produce final concentrations of 3.4725, 6.945, 34.725, 69.45, 347.25, 694.5, and 1389 ng/mL for psoralen and 3.81, 7.62, 38.1, 76.2, 381, 762, and 1524 ng/mL for isopsoralen. Similarly, quality control (QC) samples were prepared in the same manner at concentrations of 6.945, 69.45, and 1111.2 ng/mL for psoralen and 7.62, 76.2, and 1219.2 ng/mL for isopsoralen. All solutions were stored at 4°C refrigerator and brought to room temperature before use.

### 2.5. Method Validation

#### 2.5.1. Specificity

The specificity of the method was investigated by comparing chromatograms of blank kidney homogenate sample, blank kidney homogenate sample spiked with standard solution and IS, and treated kidney tissue homogenate sample.

#### 2.5.2. Linearity and Quantification

Various concentrations of psoralen and isopsoralen calibration standard solutions with IS (30 ng/mL) were added to blank tissue treated as tissue samples and assayed by UHPLC-MS/MS system. The calibration curve was established via 1/*x* ^2^ weighted linear least-squares regression model. The lower limit of quantification (LLOQ) had the lowest concentrations with signal-to-noise ratio *⩾* 10, evaluated by analyzing samples in six replicates. The lower limit of detection (LLOD) was defined as the amount that could be detected with a signal-to-noise ratio *⩾* 3.

#### 2.5.3. Precision and Accuracy

Accuracy and precision of the method were determined by repeat analyses of QC and LLOQ samples. The intraday precision and accuracy of the method were assessed by determining QC samples six times within a single day, while the interday precision and accuracy were estimated by determining QC samples over three consecutive days.

#### 2.5.4. Recovery and Matrix Effects

The extraction recovery was calculated by comparing the peak areas of extracted QC samples with peak areas of psoralen and isopsoralen reference standard solutions. Matrix effects of the method were determined by comparing peak areas of blank plasma extracts spiked with standard samples with peak areas of neat standard solution.

#### 2.5.5. Stability

The stability of analytes in tissues was evaluated by measuring three concentrations of the QC samples (*n* = 6) under different conditions. The short-term stability was investigated by exposing the QC samples at room temperature for 4 h. The long-term stability was assessed after storing the QC samples at −20°C for 30 days. Freezing-thawing stability was determined after QC samples underwent three freezing-thawing cycles by freezing at −20°C freezer and thawing at 37°C water bath.

### 2.6. Tissue Distribution Study

A total of 72 Sprague-Dawley rats, weighing 180–220 g, half male and half female, which were provided by Experimental Animal Center of Nanjing University of Chinese Medicine, were randomly divided into 2 groups, with 36 rats in each group. These rats were housed with free access to laboratory food and water and deprived of food for 12 h with water available prior to the experiment. These two groups of rats were orally given raw and salt-processed scurfpea fruit decoction at the same dose of 2.0 mL/200 g of body weight, separately. Heart, liver, spleen, lung, kidney, ovary, and testis were collected after dosing at 5, 10, 150, 360, 480, and 720 min (each time point sacrificed 6 rats for each group, half male and half female) and then immediately washed with 0.9% normal saline solution and blotted up with filter paper and then stored at −20°C for further analysis.

Before analyzing, the collected tissues were homogenized in 2-fold of 0.9% normal saline solution separately. For each sample, 10 *μ*L IS and 300 *μ*L acetonitrile were added to 100 *μ*L tissue homogenate. The mixtures were vortexed for 3 min and centrifuged at 4000 rpm for 5 min. Supernatant of each sample was transferred into a 1.5 mL Eppendorf tube individually and centrifuged at 12000 rpm for 3 min one more time. Finally, 200 *μ*L of the supernatant was taken for UHPLC-MS/MS analyzing.

### 2.7. Targeting Efficiency Evaluation

The purpose of salt-processing of scurfpea fruit in TCM theory is somewhat similar to modern drug target-delivery theory. Considering this, in this research, relative uptake efficiency (RUE), RC_max_, and relative targeting efficiency (RTE) were utilized to investigate the effect of salt-processing on the tissue distribution of psoralen and isopsoralen. The relevant parameters were calculated according to the following equations [17, 18]:

(1) RUE=AUCsalt-processedAUCcrude,RCmax=Cmax salt-processedCmax crude,RTE=AUCsalt-processed/AUCsum−AUCcrude/AUCsumAUCcrude/AUCsum,AUCsum=AUCheart+AUCliver+AUCspleen+AUClung+AUCkidney+AUCovarytestis.

In these equations, AUC_sum_ involves the sum of AUC of all tissues in salt-processed and crude groups, respectively.

### 2.8. Data Analysis

The pharmacokinetic parameters were obtained by using Drug and Statistics 2.0 (DAS 2.0) software. All of the statistics analyses were conducted by using SPSS 16.0 for Windows.

As the contents of psoralen and isopsoralen in crude and salt-processed* P. corylifolia* L. extracts were not the same, in order to eliminate the effect of different dosage of psoralen and isopsoralen of crude and salt-processed* P. corylifolia* L. groups, pharmacokinetic parameter AUC of psoralen and isopsoralen of salt-processed* P. corylifolia* L. group was calculated before use by the following equation:

(2) the treated salt-processed group=salt-processed groupcontents of salt-processed/contents of crude.

## 3. Results

### 3.1. Method Optimization

#### 3.1.1. Specificity

**Figure 2:**
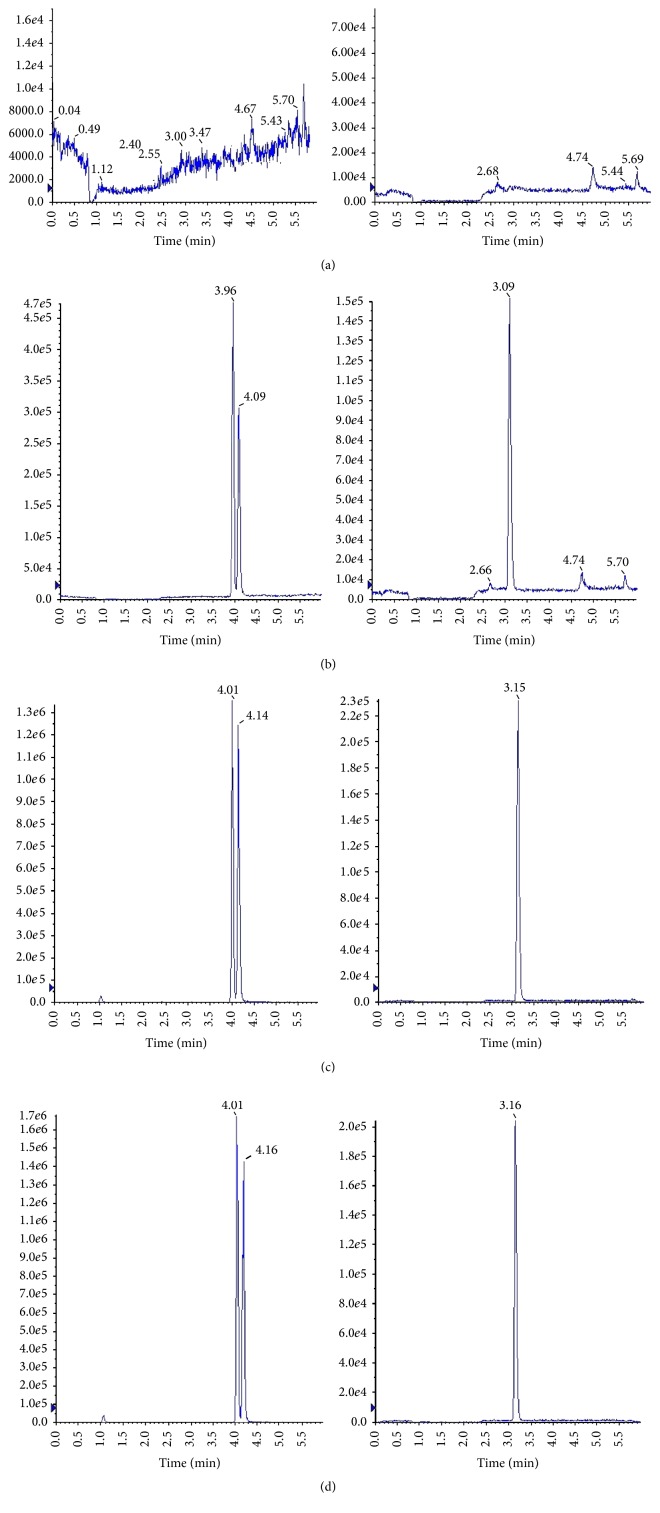
SIM chromatograms of psoralen, isopsoralen, and IS in rats plasma. (a) blank tissue; (b) blank tissue spiked with psoralen, isopsoralen, and IS; (c) kidney tissue sample of oral administration of crude scurfpea fruit; (d) kidney tissue sample of oral administration of salt-processed scurfpea fruit.

The HPLC profiles, which were obtained from blank kidney homogenate sample, blank kidney homogenate sample spiked with standard solution and IS, and kidney tissue homogenate sample at 360 min after oral administration of raw or processed scurfpea fruit, were shown in Figure 2. Under the chromatographic conditions described above, psoralen and isopsoralen were eluted with retention time of 4.01 and 4.14 min of crude group and 4.01 and 4.16 min of salt-processed group, respectively. No endogenous peaks were observed in blank kidney samples or other 3 groups.

#### 3.1.2. Linearity and Sensitivity

The equations for the calibration curves, linear range, and LLOQ of psoralen and isopsoralen were presented in Table 1. Results showed that calibrations curves were linear and acceptable. The ranges of concentrations and limits were suitable for the tissue distribution study of psoralen and isopsoralen.

#### 3.1.3. Precision and Accuracy

The precision and accuracy of the assay were evaluated by QC samples at low, medium, and high concentrations. The precision and accuracy of psoralen and isopsoralen were shown in Table 2. For psoralen, the intraday and interday precision values were not more than 9.85% and 7.09%, respectively. For isopsoralen, the intraday and interday precision values were within the range between 6.17% and 5.56%. Results indicated that the accuracy and precision of the method were within acceptable thresholds.

#### 3.1.4. Recovery

The extraction recoveries were presented in Table 2. From Table 2, QC sample at low, medium, and high concentrations was well within 68.93%–81.40% for psoralen and 67.89%–81.50% for isopsoralen, respectively. The RSD of extraction recoveries were less than 4.74% for psoralen and 6.50% for isopsoralen, correspondently.

#### 3.1.5. Stability

The results of stability under different storage conditions were presented in Table 3. Results manifested that samples were stable under various experimental conditions.

### 3.2. Contents of Psoralen and Isopsoralen in Scurfpea Fruit Extracts

The contents of psoralen and isopsoralen of crude and salt-processed scurfpea fruit extracts were quantified by using external standard method. Result showed that the concentration of psoralen and isopsoralen was 1034 ng/mL and 924 ng/mL in crude herb solution and 971.6 ng/mL and 832.4 ng/mL in salt-processed herb solution correspondently.

### 3.3. Tissue Distribution Studies

**Figure 3:**
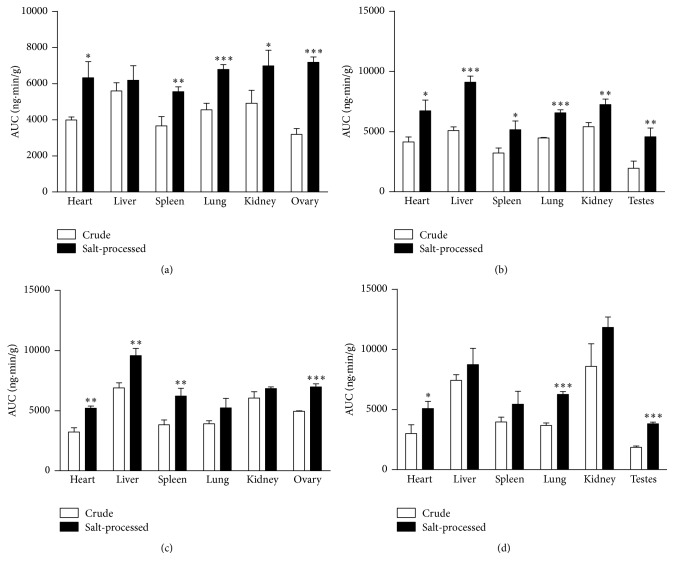
The AUC profiles of main ingredient of scurfpea fruit in rat tissues. (a) Psoralen in female rat; (b) psoralen in male rat; (c) isopsoralen in female rat; (d) isopsoralen in male rat. Salt-processed group compared to crude group; ^*∗*^ *p* < 0.05; ^*∗∗*^ *p* < 0.01; ^*∗∗∗*^ *p* < 0.001.

A validated UHPLC-MS/MS method was applied to investigate psoralen and isopsoralen in rat tissues after oral administration of raw and salt-processed scurfpea fruit. By using DAS 2.0 to process the tissue distribution data, the AUC figures were obtained. The AUC figures of psoralen and isopsoralen were displayed in Figure 3. Figures 3(a) and 3(c) presented the AUC of psoralen and isopsoralen in female rats, respectively, with comparison of administrated crude and salt-processed scurfpea fruit decoction. It is shown that the highest increment of psoralen after salt-processing was in lung and ovary (*p* < 0.001), followed by spleen (*p* < 0.01), kidney, and heart (*p* < 0.05), while for isopsoralen the highest increment was in ovary (*p* < 0.001), followed by spleen and heart (*p* < 0.01). Figures 3(b) and 3(d) presented the AUC of psoralen and isopsoralen in male rats, respectively, with comparison of administrated crude and salt-processed scurfpea fruit decoction. It is shown that psoralen of salt-processed group in all tissues is significantly greater than that of crude group. For isopsoralen, the remarkable increase was noted in testes, lung (*p* < 0.001), and heart (*p* < 0.01). In brief, the AUC of psoralen and isopsoralen of salt-processed group was higher than that of crude group in all tissues, which indicated that salt-processing of scurfpea fruit would enhance the bioavailability of both components.

### 3.4. Targeting Efficiency Evaluation

In general, targeting efficiency was determined by comparing the drug concentration and retaining time in the target site to those of the nontarget sites, and the area under concentration-time curve (AUC), maximum concentration (*C*_max_), relative uptake ratio (RUE), relative *C*_max_ (RC_max_), relative targeting ratio (RTE), and targeting index could be used for targeting evaluation [19]. Since there are some correlations in these parameters, RUE, RC_max_, and RTE were selected to evaluate the targeting organs after salt-processing of scurfpea fruit in this paper.

#### 3.4.1. RUE as the Index

**Figure 4:**
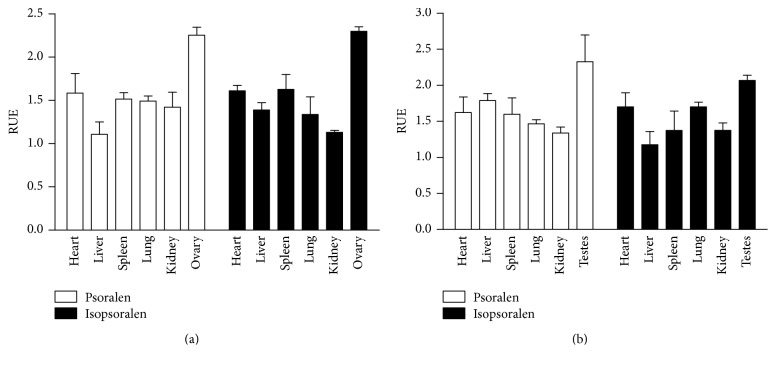
The RUE profiles of psoralen and isopsoralen in male and female rat tissues. (a) Psoralen and isopsoralen in female rat; (b) psoralen and isopsoralen in male rat.

RUE is an index for targeting evaluation, and it could reflect the effect of salt-processing on the distribution of psoralen and isopsoralen in different organs. The results of salt-processing on the RUE of psoralen and isopsoralen are shown in Figure 4. In female group, the RUE of psoralen and isopsoralen in all tissues was greater than 1. The tendency of psoralen in various tissues was ranked as follows: ovary > heart > spleen > lung > kidney > liver, while the tendency of isopsoralen was ranked as follows: ovary > spleen > heart > liver > lung > kidney. Similarly, in male group, the RUE of psoralen and isopsoralen in all tissues was also greater than 1. RUE of psoralen in the tissues presented the following tendency: testis > heart > spleen > kidney > lung > liver; the RUE of isopsoralen was ranked as follows: testis > spleen > heart > liver > kidney > lung. In general, the maximum RUE of psoralen and isopsoralen was in generative organ, followed by heart and spleen.

#### 3.4.2. RC_max_ as the Index

**Figure 5:**
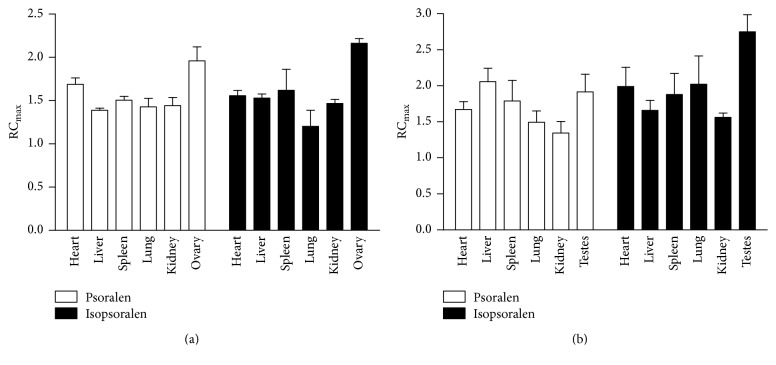
RC_max_ profiles of psoralen and isopsoralen in male and female rat tissues. (a) Psoralen and isopsoralen in female rat; (b) psoralen and isopsoralen in male rat.

*C*_max_ is a parameter that can reflect the distribution extent and can also be used as an index of targeting. RC_max_ is the ratio of *C*_max_ between raw group and salt-processed group in various organs. It could reflect the distribution change after salt-processing. As shown in Figure 5(a), for female rats, RC_max_ of psoralen and isopsoralen in all tissues was greater than 1. RC_max_ of psoralen was ranked as follows: ovary > heart > spleen > lung > kidney > liver. The RUE value of isopsoralen showed similar order, with maximum RC_max_ of 2.164 in ovary, followed by 1.620 in spleen and 1.557 in heart. In Figure 5(b), for male rats, all RC_max_ was greater than 1; for psoralen, maximum RC_max_ was 2.059 in liver, followed by 1.916 in testes, 1.788 in spleen, and 1.672 in heart; for isopsoralen, maximum RC_max_ was 2.7293 in testes, 2.022 in lung, 1.992 in heart, and 1.878 in spleen. RC_max_ showed that the salt-processing significantly enhanced the targeting effect of both components to generative organs, followed by heart and spleen.

#### 3.4.3. RTE as the Index

**Figure 6:**
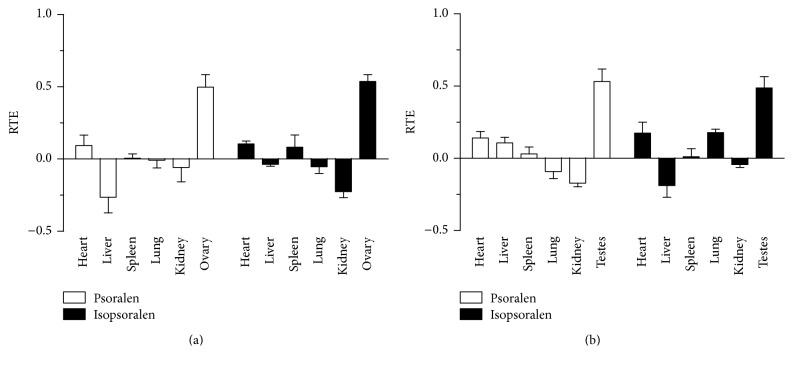
The RTE profiles of psoralen and isopsoralen in male and female rat tissues. (a) Psoralen and isopsoralen in female rat; (b) psoralen and isopsoralen in male rat.

RTE is a drug distribution ratio by comparing the salt-processed group to that of the control group. When RTE is above zero, it will indicate a targeting enhancing effect; otherwise, it will indicate a targeting weakening effect. The results of RTE are shown in Figure 6. Similar to the result of RUE and RC_max_, in female group, the maximum RTE of psoralen was 0.497 in ovary, followed by 0.093 in heart and 0.005 in spleen; RTE in other organs was below zero; for isopsoralen, the result was similar to the RTE of psoralen; the maximum RTE was 0.537 in ovary, followed by heart and spleen; the RTE of psoralen and isopsoralen in kidney and lung was less than zero. In male group, the maximum RTE of psoralen was 0.532 in testes, followed by 0.141 in heart, 0.107 in liver, and 0.030 in spleen; the RTE of isopsoralen was 0.487 in testes, 0.179 in lung, 0.175 in heart, and 0.010 in spleen; the RTE of psoralen and isopsoralen in kidney was lower than zero.

## 4. Discussion

By comparing the AUC figures, it was obviously shown that, after salt-processing, the distribution of psoralen and isopsoralen in all organs, including heart, liver, spleen, lung, kidney, ovary, and testis, was increased, and the highest increment happened in generative organs. As the contents of psoralen and isopsoralen of salt-processed group have been unified the same as the crude group in data analyse, it manifested that the increment of psoralen and isopsoralen distribution in relative organs after salt-processing was not due to the concentration difference. The higher absorption rate of the 2 components from salt-processed group may be induced by the high osmotic pressure caused by salt; however, the mechanism of it should be further investigated.

The RUE, RC_max_, and RTE values demonstrated that salt-processing significantly enhanced the targeting effect of psoralen and isopsoralen to generative organs, heart and spleen. This is in accordance with the channel tropism theory of TCM, in which the scurfpea fruit is attributed to spleen meridian and kidney meridian. Spleen meridian was related to spleen and stomach and so forth [20]. The kidney meridian includes kidney and generative organs, such as ovary in female and testes in male. It was related to the hypothalamus-pituitary-gonad-axis [21]. The results showed that the RUE, RC_max_, and RTE values in generative organs were much greater than heart and spleen, which indicated that psoralen and isopsoralen exhibited targeting effect to generative organs after salt-processing. It was reported that the estrogen-like activity of psoralen and isopsoralen was caused by their characters acting as estrogen receptor *α* agonists [22].

As the RTE value of psoralen and isopsoralen in kidney was less than zero, this means the targeting effect only focused on generative organs of the kidney meridian. Huang reported that salt-processed* Alpiniae oxyphyllae* could target its effective components on kidney and enhance the polyuria arrest effect [23]. Salt-processing is a widely used herb processing technology in TCM. It may be utilized to process various herbs with different functions. According to TCM theory, channel tropism of an herb may involve several relative organs. Salt-processing of an herb may enhance the activity of its components to act on one individual organ or several relevant organs simultaneously, depending on herb's property. It desires further exploration to enrich our knowledge on salt-processing mechanism.

## 5. Conclusion

This study firstly revealed the fact that salt-processed scurfpea fruit could enhance the generative organs targeting effect of its two main active components, namely, psoralen and isopsoralen. A sensitive and accurate UHPLC-MS-MS method was established for the simultaneous determination of psoralen and isopsoralen in rat tissues. Results provided a firm basis to investigate the material base of the clinical efficacy of TCM and also will be helpful to explain the effect of salt-processing on guiding drugs to kidney meridian.

## Figures and Tables

**Table 1:** Calibration curves, linear range, and LLOQ of psoralen and isopsoralen.

Compound	Tissue	Calibration curves	Correlation coefficient (*r* ^2^)	Linear range (ng/mL)	LLOQ (ng/mL)
Psoralen	Heart	*y* = 0.87079*x* + 0.12692	*r* = 0.9953	3.4725–1389	3.4725
	Liver	*y* = 0.93952*x* + 0.08786	*r* = 0.9980	3.4725–1389	3.4725
	Spleen	*y* = 0.85143*x* + 0.08797	*r* = 0.9995	3.4725–1389	3.4725
	Lung	*y* = 1.03681*x* + 0.09524	*r* = 0.9956	3.4725–1389	3.4725
	Kidney	*y* = 1.00241*x* + 0.09995	*r* = 0.9984	3.4725–1389	3.4725
	Ovary	*y* = 0.90176*x* + 0.11619	*r* = 0.9973	3.4725–1389	3.4725
	Testis	*y* = 0.89652*x* + 0.16793	*r* = 0.9985	3.4725–1389	3.4725

Isopsoralen	Heart	*y* = 0.90888*x* + 0.37652	*r* = 0.9972	3.81–1524	3.81
	Liver	*y* = 1.12790*x* + 0.27447	*r* = 0.9949	3.81–1524	3.81
	Spleen	*y* = 1.01521*x* + 0.17162	*r* = 0.9991	3.81–1524	3.81
	Lung	*y* = 1.09015*x* + 0.32804	*r* = 0.9966	3.81–1524	3.81
	Kidney	*y* = 1.10237*x* + 0.41405	*r* = 0.9986	3.81–1524	3.81
	Ovary	*y* = 1.00497*x* + 0.31223	*r* = 0.9973	3.81–1524	3.81
	Testis	*y* = 0.95115*x* + 0.35855	*r* = 0.9971	3.81–1524	3.81

**Table 2:** Intra-assay and interassay precision, accuracy, and extract recovery of psoralen and isopsoralen in rat tissues (*n* = 3 for sexual organs; *n* = 6 for other tissues).

Compound	Tissue	Concentrations	Intraday		Interday		Extract recovery
			Precision	Accuracy	Precision	Accuracy	
		(ng/mL)	(%)	(%)	(%)	(%)	(%, mean ± SD)
Psoralen	Heart	6.945	1.38	−0.85	1.99	−1.40	74.35 ± 1.41
		69.45	2.27	−0.03	2.20	−0.92	77.44 ± 3.53
		1111.2	4.15	1.13	2.23	−0.70	78.45 ± 3.59
	Liver	6.945	3.47	3.34	3.41	4.41	76.48 ± 3.15
		69.45	2.43	4.47	2.04	4.70	78.90 ± 3.27
		1111.2	7.09	−0.40	6.31	−0.17	77.86 ± 2.39
	Spleen	6.945	1.87	2.78	9.53	2.16	81.25 ± 2.96
		69.45	1.76	−9.34	9.85	2.16	77.91 ± 4.10
		1111.2	3.28	2.20	2.11	4.18	73.67 ± 3.57
	Lung	6.945	1.93	4.11	1.01	0.07	74.69 ± 3.97
		69.45	1.41	7.66	3.21	2.09	75.84 ± 2.07
		1111.2	2.57	−17.28	3.42	3.98	79.16 ± 1.73
	Kidney	6.945	1.77	−1.11	1.45	0.16	76.80 ± 4.74
		69.45	1.59	0.62	1.24	−0.08	76.43 ± 2.98
		1111.2	7.43	0.29	6.50	−3.02	75.81 ± 3.74
	Ovary	6.945	0.53	−0.31	1.06	0.20	69.72 ± 2.80
		69.45	0.69	0.76	0.41	0.45	68.93 ± 3.17
		1111.2	0.57	−0.29	0.42	0.67	81.40 ± 3.01
	Testis	6.945	1.01	0.45	1.40	0.56	76.54 ± 3.08
		69.45	0.91	0.52	1.09	0.68	76.46 ± 2.34
		1111.2	2.75	3.85	2.87	1.15	76.06 ± 2.96

Isopsoralen	Heart	7.62	1.39	0.98	1.62	0.44	71.78 ± 3.69
		76.2	1.30	1.67	0.74	0.08	76.91 ± 4.23
		1219.2	2.38	2.16	2.45	−0.49	78.94 ± 2.95
	Liver	7.62	3.78	−1.34	4.34	−1.68	72.48 ± 1.04
		76.2	2.54	5.50	5.34	1.49	76.95 ± 2.57
		1219.2	4.34	−9.42	5.56	−4.67	76.24 ± 3.66
	Spleen	7.62	2.43	1.23	1.60	−0.29	71.62 ± 3.45
		76.2	3.60	−0.01	1.80	2.05	75.35 ± 3.57
		1219.2	4.17	−5.59	6.24	−1.11	77.84 ± 2.59
	Lung	7.62	3.79	−1.34	4.34	−1.68	78.73 ± 5.49
		76.2	2.54	5.49	5.34	1.49	78.80 ± 6.08
		1219.2	4.34	−9.42	5.56	−4.67	81.50 ± 6.50
	Kidney	7.62	1.01	0.90	1.27	0.62	80.01 ± 2.41
		76.2	1.57	1.66	1.04	−0.34	79.48 ± 2.92
		1219.2	6.17	−6.48	5.47	−7.02	73.69 ± 0.51
	Ovary	7.62	1.34	0.23	2.05	0.27	68.71 ± 1.45
		76.2	1.20	0.58	0.87	−0.57	67.89 ± 2.15
		1219.2	5.13	−0.26	6.27	−1.18	77.47 ± 5.47
	Testis	7.62	2.13	−0.17	1.23	−0.22	74.05 ± 2.46
		76.2	1.37	0.19	1.06	0.32	75.25 ± 2.36
		1219.2	2.91	3.79	3.31	3.13	77.18 ± 2.04

**Table 3:** Stability of psoralen and isopsoralen in rat tissues. (*n* = 3 for sexual organs; *n* = 6 for other tissues).

Compound	Tissues	Spiked concentrations	Freeze-thaw stability		Short-term stability		Long-term stability	
		(ng/mL)	Mean ± SD	RE (%)	Mean ± SD	RE (%)	Mean ± SD	RE (%)
Psoralen	Heart	6.945	6.80 ± 0.11	−2.16	6.76 ± 0.21	−2.73	6.82 ± 0.14	−1.83
		69.45	68.23 ± 0.91	−1.76	67.11 ± 2.25	−3.38	70.42 ± 1.17	1.40
		1111.2	1062.25 ± 83.85	−4.41	1051.78 ± 23.11	−5.35	1131.52 ± 10.56	1.83
	Liver	6.945	7.07 ± 0.37	1.82	7.12 ± 0.08	2.59	7.10 ± 0.04	2.25
		69.45	74.10 ± 1.39	6.69	71.58 ± 2.71	3.06	70.66 ± 1.09	1.74
		1111.2	1052.56 ± 54.21	−5.28	1150.08 ± 44.05	3.50	1097.14 ± 65.50	−1.27
	Spleen	6.945	6.91 ± 0.13	−0.49	6.83 ± 0.14	−1.71	7.07 ± 0.06	1.80
		69.45	68.54 ± 0.22	−1.30	67.94 ± 2.24	−2.15	67.60 ± 0.31	−2.66
		1111.2	1049.60 ± 16.07	−5.54	1130.31 ± 54.07	1.72	1114.40 ± 70.06	0.29
	Lung	6.945	7.16 ± 0.12	3.05	7.40 ± 0.10	−5.27	7.34 ± 0.30	5.75
		69.45	73.80 ± 0.02	6.27	66.44 ± 0.98	−4.34	74.02 ± 1.78	6.59
		1111.2	1008.84 ± 53.80	−9.21	651.02 ± 7.32	−41.41	915.37 ± 75.71	−17.62
	Kidney	6.945	6.66 ± 0.32	−4.04	6.97 ± 0.12	0.38	6.84 ± 0.02	−1.48
		69.45	70.30 ± 1.11	1.23	69.25 ± 2.06	−0.30	70.24 ± 0.41	1.14
		1111.2	1038.70 ± 18.58	−6.52	1082.02 ± 48.19	−2.63	1109.80 ± 78.26	−1.26
	Ovary	6.945	6.88 ± 0.18	−0.96	6.96 ± 0.05	0.17	7.04 ± 0.11	1.39
		69.45	69.25 ± 2.59	−0.29	69.66 ± 1.22	0.30	70.11 ± 0.74	0.96
		1111.2	1038.33 ± 17.84	−6.56	1130.92 ± 32.55	1.77	1099.28 ± 43.85	−1.07
	Testis	6.945	6.87 ± 0.20	−1.12	6.48 ± 0.11	−6.71	6.78 ± 0.17	−2.31
		69.45	68.19 ± 0.18	−1.82	69.82 ± 1.90	0.53	70.77 ± 7.55	1.90
		1111.2	1077.27 ± 79.52	−3.05	1086.87 ± 41.36	−2.19	1050.69 ± 77.20	−5.45

Isopsoralen	Heart	7.62	7.53 ± 0.26	−1.23	7.26 ± 0.09	−4.69	7.46 ± 0.17	−2.16
		76.2	77.58 ± 0.85	1.81	75.01 ± 1.42	−1.56	75.79 ± 0.81	−0.54
		1219.2	1108.20 ± 75.09	−9.10	1166.75 ± 67.08	−4.30	1284.62 ± 15.80	5.37
	Liver	7.62	7.08 ± 0.05	−5.17	7.38 ± 0.10	−3.24	7.20 ± 0.07	−4.60
		76.2	72.31 ± 0.24	−5.11	76.49 ± 1.17	0.38	71.92 ± 0.85	−5.61
		1219.2	1126.26 ± 25.38	−5.83	1154.34 ± 45.06	−5.32	1120.07 ± 48.38	−8.13
	Spleen	7.62	7.75 ± 0.12	1.73	7.66 ± 0.11	0.56	7.58 ± 0.10	−0.59
		76.2	76.86 ± 0.98	0.86	76.88 ± 0.63	0.90	78.33 ± 1.21	2.79
		1219.2	1140.32 ± 55.99	−6.47	1100.51 ± 67.67	−9.73	1143.83 ± 87.36	−6.18
	Lung	7.62	7.51 ± 0.39	−1.46	7.54 ± 0.09	−1.03	7.24 ± 0.21	−4.95
		76.2	79.23 ± 1.79	3.98	81.08 ± 0.93	0.06	79.02 ± 3.35	3.70
		1219.2	1187.77 ± 56.03	−2.58	1135.93 ± 38.04	−7.01	1163.82 ± 76.16	−5.38
	Kidney	7.62	7.62 ± 0.05	−0.02	7.61 ± 0.24	−0.13	7.40 ± 0.35	−2.85
		76.2	76.28 ± 1.53	0.11	74.53 ± 4.50	−2.19	76.85 ± 0.14	0.85
		1219.2	1111.45 ± 55.37	−4.66	1185.88 ± 96.40	−2.73	1231.28 ± 71.66	0.99
	Ovary	7.62	7.55 ± 0.11	−0.87	7.57 ± 0.28	−0.68	7.67 ± 0.19	0.67
		76.2	77.38 ± 0.89	1.55	76.55 ± 1.16	0.46	77.47 ± 1.03	1.67
		1219.2	1164.91 ± 55.17	−4.45	1250.56 ± 29.56	2.57	1227.01 ± 41.73	0.64
	Testis	7.62	7.77 ± 0.27	1.92	7.32 ± 0.33	−3.95	7.51 ± 0.32	−1.44
		76.2	76.07 ± 1.85	−0.18	76.80 ± 1.56	0.85	76.17 ± 3.56	−0.04
		1219.2	1241.11 ± 20.93	3.95	1180.85 ± 11.49	−4.15	1104.70 ± 68.05	−7.59

## Abbreviations

AUC: Area under the curve
CE: Collision energy
CUR: Curtain gas
CXP: Collision exit potential
DP: Declustering potential
EP: Entrance potential
ESI: Information-dependent acquisition
IS: Internal standard
LLOD: The lower limit of detection
LLOQ: The lower limit of quantitation
MRM: Multiple reaction monitoring
QC: Quality control
RC_max_: The maximum relative concentration
RTE: Relative targeting efficiency
RUE: Relative uptake efficiency
SD: Sprague-Dawley
TCM: Traditional Chinese medicine
TEM: Turbo spray temperature
UHPLC-MS/MS: Ultrahigh performance liquid chromatography tandem mass spectrometry.
